# Drug tolerant persister cell plasticity in cancer: a revolutionary strategy for more effective anticancer therapies

**DOI:** 10.1038/s41392-024-01891-4

**Published:** 2024-08-14

**Authors:** Jun He, Zejing Qiu, Jingjing Fan, Xiaohong Xie, Qinsong Sheng, Xinbing Sui

**Affiliations:** 1 Department of Medical Oncology, the Affiliated Hospital of Hangzhou Normal University, Hangzhou Normal University, Hangzhou, Zhejiang 311121 China; 2 Key Laboratory of Elemene Class Anti-Cancer Chinese Medicines of Zhejiang Province, Hangzhou Normal University, Hangzhou, Zhejiang 311121 China; 3 Department of Breast Surgery, The First Affiliated Hospital of Zhejiang Chinese Medical University, Hangzhou, Zhejiang China; 4 Department of Colorectal Surgery, The First Affiliated Hospital, School of Medicine, Zhejiang University, Hangzhou, Zhejiang China

**Keywords:** Cancer therapy, Tumour immunology, Cancer metabolism

## Abstract

Non-genetic mechanisms have recently emerged as important drivers of anticancer drug resistance. Among these, the drug tolerant persister (DTP) cell phenotype is attracting more and more attention and giving a predominant non-genetic role in cancer therapy resistance. The DTP phenotype is characterized by a quiescent or slow-cell-cycle reversible state of the cancer cell subpopulation and inert specialization to stimuli, which tolerates anticancer drug exposure to some extent through the interaction of multiple underlying mechanisms and recovering growth and proliferation after drug withdrawal, ultimately leading to treatment resistance and cancer recurrence. Therefore, targeting DTP cells is anticipated to provide new treatment opportunities for cancer patients, although our current knowledge of these DTP cells in treatment resistance remains limited. In this review, we provide a comprehensive overview of the formation characteristics and underlying drug tolerant mechanisms of DTP cells, investigate the potential drugs for DTP (including preclinical drugs, novel use for old drugs, and natural products) based on different medicine models, and discuss the necessity and feasibility of anti-DTP therapy, related application forms, and future issues that will need to be addressed to advance this emerging field towards clinical applications. Nonetheless, understanding the novel functions of DTP cells may enable us to develop new more effective anticancer therapy and improve clinical outcomes for cancer patients.

## Introduction

Resistance to anticancer drug therapy, especially first-generation targeted therapy products, is a critical bottleneck in comprehensive anticancer treatment. For example, patients with lung cancer treated with Gefitinib, a first-generation epidermal growth factor receptor (EGFR) inhibitor, typically develop resistance after 9 to 15 months of progression-free survival.^1^ Genetic mechanisms, such as mutations in the T790M gene,^2,3^ have been partially attributed to the development of drug resistance. However, evidence is increasingly pointing towards non-genetic mechanisms as playing a critical role in resistance development, with the presence of DTP cells being a key factor.^4^

DTP cells refer to a subgroup of cancer cells with distinct biological characteristics^5^ and internal mechanisms,^6^ allowing them to resist initial anticancer treatment. Unlike specific or characteristic cancer cells,^7^ DTP cells persist in the anticancer drug environment, exhibiting significant variances from typical cancer cells in aspects such as cell-cycle, micro-environment regulation, and metabolism. These differences confer upon them the ability to endure and subsequently contribute to cancer recurrence and metastasis. Currently, research on the mechanism of DTP formation and drug resistance has primarily been conducted through cellular tests,^8–12^ animal tests,^13–16^, and mathematical models.^13^ Some studies have likewise mentioned the potential of anti-DTP treatments.^8,17,18^ However, no clinical studies have yet confirmed the existence of DTP cells or the effectiveness of anti-DTP treatments following anticancer therapy in humans. The lack of quantifiable understanding of DTP and the unclear direction of its potential clinical treatment hinders further exploration. This review provides a comprehensive characterization of the biology and basic mechanism of action of DTP cells, along with descriptions of potential therapies for anti-DTP. Additionally, it extends the direction of future development and application, providing ideas and guidelines to promote this emerging field towards clinical application.

## Proposed DTP: Evolution of research developments on DTP cells

### Chronicle of DTP cells

**Fig. 1:**
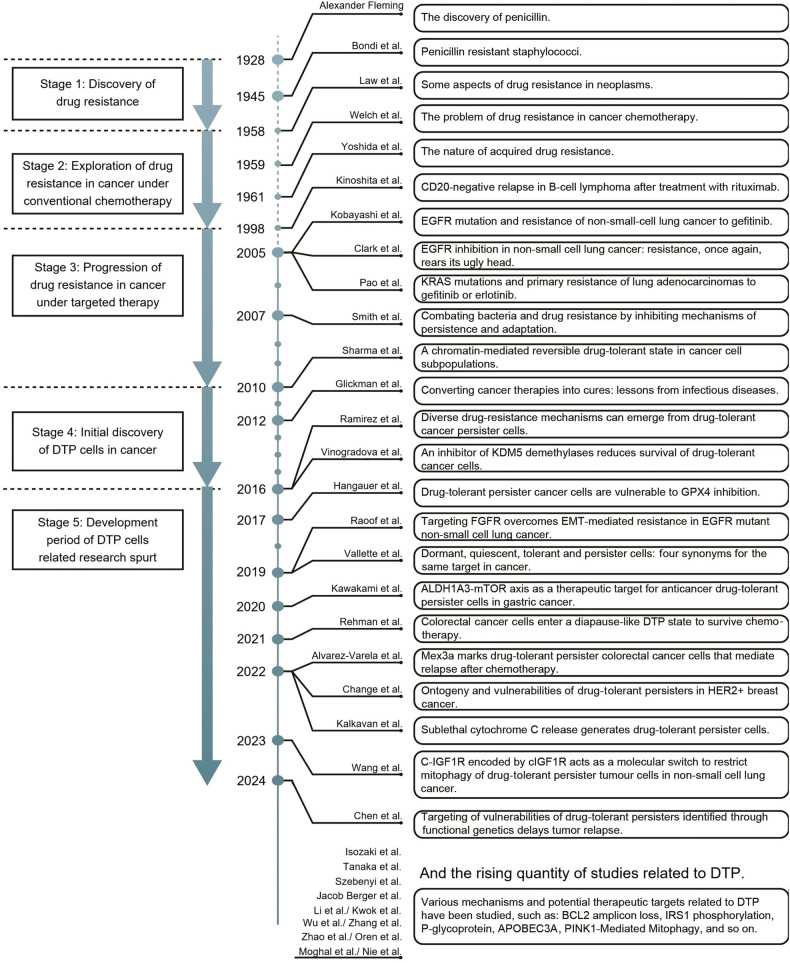
The historical timeline of the DTP concept from the inception to the present peak. At the initial beginning, the discovery of antibiotics and the drug resistance phenomenon^290^ was the first critical period of human cognitive resistance (Stage 1). After recognizing cancer and traditional chemical therapy, the frequently limited effectiveness of treatment has led researchers to observe the widespread problem of drug resistance in anticancer drug therapy^291–293^ (Stage 2). The newly developed targeted therapy also owns the problem of drug resistance in anticancer therapy,^294–297^ which further deepens the thinking on the root nature of cancer drug resistance^24^ (Stage 3). With the initial observation of a chromatin-mediated reversible drug-tolerant state in the targeted therapy of non-small cell lung cancer,^32,112,298^ the concept of DTP has been proposed and its possibility has been preliminarily verified (Stage 4). Since then, more and more researchers have used the concept of DTP cell to bring several studies on its mechanism^6,7,13,15,16,96,111,177,238,253,288,299–301^ and treatment related^8,10,17,81,97,284,287,302,303^ analysis, and a variety of different mechanisms and possible solutions have filled the entire field of DTP cells research, and a spurt of development stage constitutes the present new period (Stage 5)

The evolution of DTP research is a part of the complex and evolving field of oncology from recognition to conjecture to confirmation (Fig. 1). Soon after the discovery of penicillin antibacterial treatment, scientists noted the phenomenon of drug resistance.^19–23^ This initial observation indicates that adaptation of treatment plays a significant role in drug resistance,^24^ advancing medical understanding in this area. With the introduction of drug therapy for cancer, researchers encountered a parallel issue of cancer cells developing resistance to anticancer drugs,^25^ echoing bacterial drug resistance. Likewise, like antimicrobials to bacteria,^26^ solely producing new drugs may be sufficient to address cancer resistance. Subsequent research has focused on the genetic mechanisms associated with gene mutations^27^ in malignant cancer cells, offering hope for addressing drug resistance. However, the unpredictable nature of mutations prevented this from becoming the definitive solution sought by researchers.^28–31^ Consequently, exploring non-genetic mechanisms emerged as a promising direction, with the discovery of a drug-resistant subpopulation by Sharma et al.^32^ attracting significant interest. The concept of DTP cells, originating from lung cancer cells,^32^ initially was considered unique lung cancer cells’ transformations rather than universal cancer cells. Nonetheless, further studies^8,33^ on DTP cells cultured from various cancer types suggested common underlying changes shared among cancer cells. Presently, the primary focus of DTP-related research lies in identifying fundamental transition rules – essential for understanding the non-genetic mechanisms of cancer drug resistance. Leveraging these principles to redefine the defense strategies in modern medicine has become a crucial research priority within the DTP field.

### Latest insight into the transition of DTP cells

**Fig. 2:**
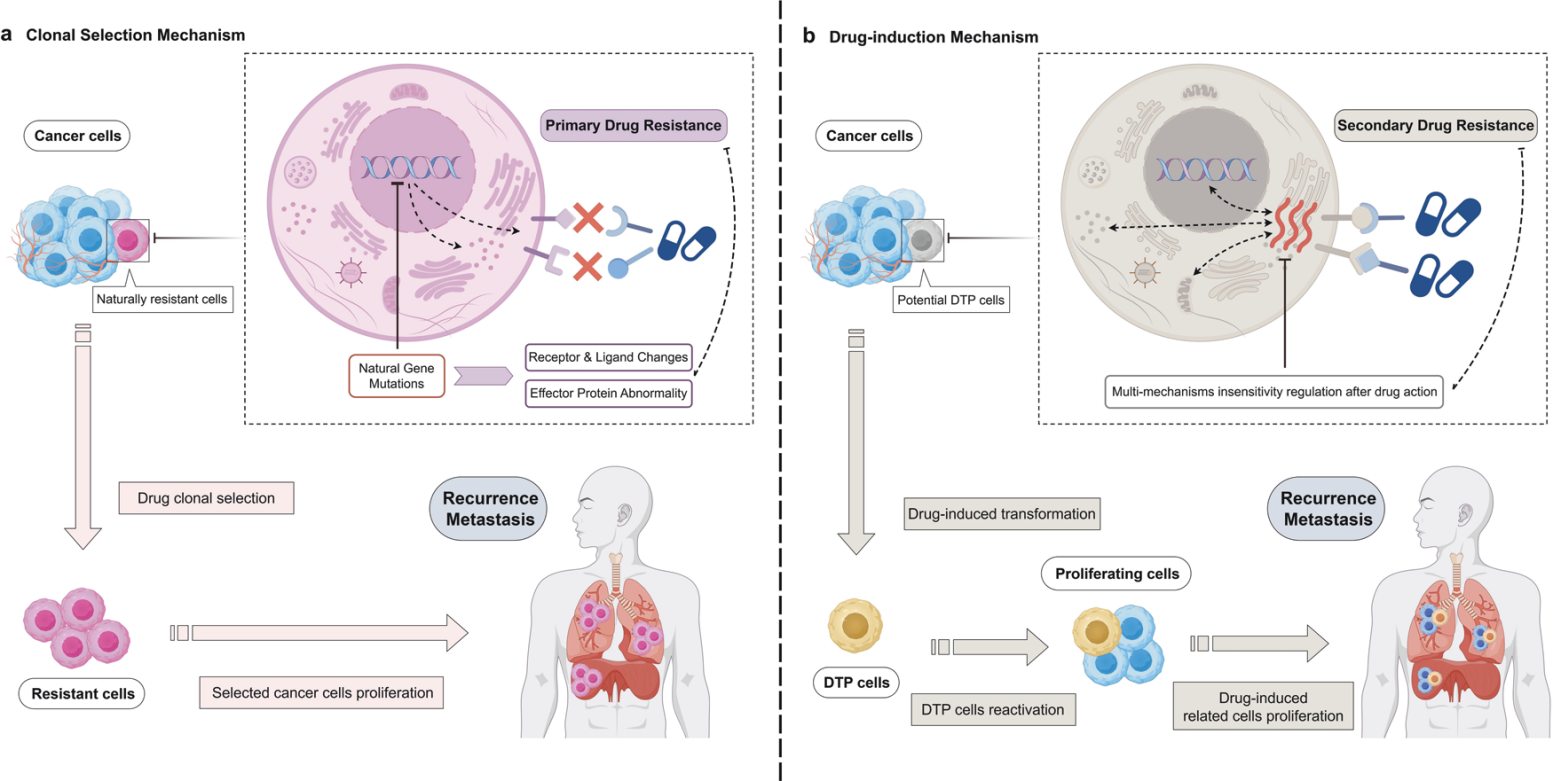
There are two primary hypotheses regarding the origin or transformation mechanisms of DTP cells and resistance to anticancer drugs: primary and secondary. Clonal Selection Mechanism (Part a): In the hypothesis of clonal selection, primary drug resistant cells with natural gene mutations, receptor/ligand changes, and effector abnormality are present within normal cancer cells and lead to cancer recurrence during external anticancer drug therapy. Drug-induction Mechanism (Part b): In the hypothesis of drug induction, the initial drug insensitive cells result from the inducing effect of anticancer drug, and lead to characteristic DTP cells during long-term drug maintenance and eventually reactivation and proliferation. Regardless of the hypothesis’s origin, DTP cells ultimately resist drug-directed cancer recurrence. (Drawn by figdraw)

In the existing blowout studies, two possible origin or transformation mechanisms of DTP cells (Fig. 2) have been discussed as relatively recent concepts.^4,34^ The first mechanism is the clonal selection mechanism hypothesis, which pertains to primary cancer resistance. The second mechanism, namely the drug induction mechanism hypothesis, is associated with secondary cancer resistance. These two mechanisms are not mutually exclusive or contradictory; however, they differ in their emphasis and thus guide divergent lines of observation in various studies.

#### Clonal selection mechanism

As the classic possible cause of cancer drug resistance,^35^ clonal selection is one possible mechanism that could lead to the emergence of DTP cells. This process is built on classical Darwinian principles,^36^ where a few persister cells may have been present before drug treatment and are subsequently selected. Due to the genomic instability of cancer cells, they may exhibit different sensitivities to drug action.^37,38^ This genomic instability could result in the emergence of DTP cells, which can then be screened for potential drug treatment.^39^ It is important to note that unstable inheritance can lead to a limited occurrence of similar phenotypes or characteristics among resistant cells, despite their common origin from the same cells, owing to genomic instability. Various studies^7,40^ have shown the presence of DTP cases lacking similar phenotypes and characteristics, while Raha et al.^41^ observed the contrary, indicating similarity in the DTP cells. Moreover, this similarity extends to holding cells resulting from the entry of cancer cells from diverse origins into the DTP cells.^42^

#### Drug induction mechanism

The second aspect is induced transformation which refers to the ability to resist treatment and withstand external drugs, environmental stresses, and other factors. In 1975, Miroslav Radman’s team discovered the existence of an inductive bacterial DNA repair/mutagenesis system (SOS response). This system is initiated in response to DNA damage, and it promotes genetic variation and reinforces bacterial resistance through the activation of transcriptional programs and genomic alterations.^43–45^ The stress-induced mutation (SIM) mechanism, which is associated with the SOS response, exists in eukaryotes^46^ and has significant implications for microbial drug resistance. Recent findings have revealed that SIMs have a role in promoting drug resistance in target organisms and can contribute to the development of resistance associated with targeted therapy. Notably, SIM has been identified as a contributing factor in acquired resistance associated with anticancer therapy, observed in diverse tumors where the therapeutic mechanism of action of the targeted drug serves as a stress-sensitive regulatory site.^47,48^ Furthermore, multiple studies^13,49–51^ have confirmed the generation of the DTP cells under drug induction, with numerous factors inducing cancer cells to transition into the DTP cells, making them a recognized potential source of drug resistance.

#### Non-genetic mechanism deserves more attention

The DTP cells discussed in this review pertain to a scenario where a small proportion of cancer cells gain resistance to prior treatments during oncological therapy while exhibiting comparable phenotypes or characteristics. It is uncertain whether drug-resistant cancer cells that lack genetic stability can be classified as DTP cells. To date, the clonal selection theory has not provided a sufficient explanation for the emergence of DTP, while the induced transformation theory does not clarify the presence of partially drug-resistant cells during the early stages of treatment.^32^ The mechanisms of both clonal selection and induced transformation are complex and contribute to the development of drug resistance in a subtle manner. Clonal selection may facilitate the initial survival of malignant cells during anticancer therapy, permitting a minority of cancer cells to acquire additional drug-resistant mutations, thereby facilitating disease progression. This ultimately leads to the survival of such cancerous cells following treatment, known as Minimal Residual Disease (MRD).^52^ Additionally, the process of transformation may cause a restricted population of cancer cells, initially vulnerable to drugs, to acquire drug resistance, sustaining this resistance during treatment and eventually resulting in tumor regrowth. The DTP cells mentioned above have been discovered in various types of cancerous tumor cells, including common lung cancer,^53^ ovarian cancer,^54^ melanoma,^55,56^ colorectal cancer^33^ etc. Interestingly, some DTP cells from various origins could manifest comparable phenotypes.^42^ Furthermore, in a separate study, Rehman et al.^13^ constructed a mathematical model concerning persister cells and ascertained that all cancer cells possess the ability to turn into DTP cells. However heritable mutations in cancer cells leading to resistance changes are commonly observed that cannot be overlooked. When researchers need to shift their attention to the non-genetic mechanisms of DTP cell components, the impact of these gene mutations can be particularly annoying. The pursuit to address this hybrid aspect has been a long-standing issue. It was not until the discovery of Mex3a^15^ and related surface antigen^57^ detection mechanisms that it became facilitative to better understand and differentiate DTP cells based on molecular probes and their unique characteristics.

The above evidence suggests that the DTP cells could be a frequently occurring stress response mechanism shared by cancer cells, so the non-genetic mechanism deserves further investigation.^58^ Of course, further studies are needed to ascertain more definitive mechanisms of DTP origin.

## Discovered DTP: Guided by the change of characteristics to explore formation of DTP cells

It is challenging to investigate and delineate the formation mechanism of DTP cells owing to the absence of direct observation and recording. Nevertheless, indirect analysis serves as a viable alternative, offering a feasible approach to look into the formation mechanism. Exploring the characteristic changes that occur before and after the formation or transformation of DTP cells represents a promising starting point in this endeavor. By offering a glimpse into the distinctive biology of DTP cells, such analysis contributes to the current, albeit limited, understanding of the enigmatic process underlying the formation of DTP cells.

### Summary of notable characteristics after DTP cells formation

**Fig. 3:**
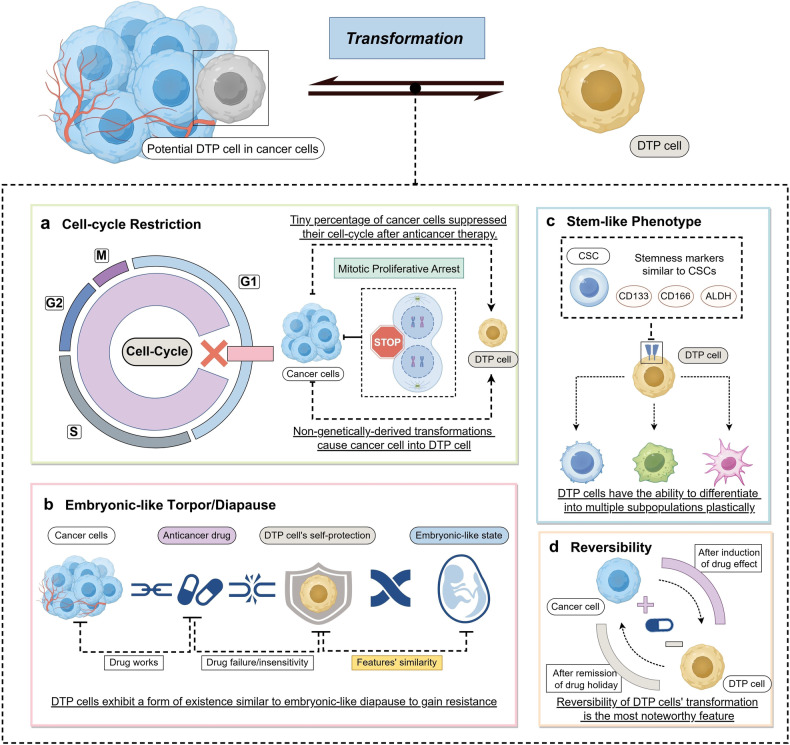
Notable characteristics of DTP cell transformation. Cycle Restriction (**a**): DTP cells concentrate in the G1 phase and do not undergo further proliferation. Embryonic-like Torpor/Diapause (**b**): Similar to organisms entering an embryonic-like state in response to external stimulus, DTP cells also exhibit a torpor/diapause state when stimulated by drugs in cancer cells. Stem-like Phenotype (Part **c**): DTP cells express some stemness markers as known cancer stem cells, such as CD133/CD166/ALDH, and have cellular plasticity. State Reversibility (**d**): DTP cells produced by cancer cell undergoing anticancer therapy can revert to a repopulating cancer cell state after discontinuation of drug therapy for a certain duration of time and subsequently exhibit DTP cells upon resuming treatment. (Drawn by figdraw)

Notably, the biological characteristics include their slow-cell-cycle properties, also known as cell-cycle restriction, their embryonic-like torpor/diapause stasis, their stem-like phenotype and plasticity, and their state reversibility. These features, which differ from those of ordinary cancer cells before transformation, are critical in shaping the nature and behavior of DTP cells (Fig. 3).

#### Cell-cycle restriction

The DTP cells, demonstrated in multiple studies, represent a state of cellular cycle quiescence or slowing down.^32,59,60^ Zhang et al.^33^ developed a model for colorectal cancer and used 5-FU treatment to control CRC growth. They observed a subset of cancerous cells that exhibited resistance to 5-FU treatment. The study identified a distinct subgroup of cancer cells referred to as DTP cells, which demonstrated significant cell-cycle related inertness. These cells displayed uncontrolled proliferation during and after 5-FU treatment. Supplementary analysis of the cell-cycle in CRC DTP cells revealed that the majority of drug-resistant cells were in the G0/G1 phase. Meanwhile, Criscione et al.^61^ investigated the impact of Osimertinib treatment on acute treatment and DTP cells in non-small cell lung cancer (NSCLC). Their study found that drug-resistant cancer cells treated with Osimertinib exhibited decreased expression of cell-cycle related genes compared to DMSO. Additionally, the study revealed that a subset of cell-cycle-related pathways and genes nearly returned to baseline levels after the removal of the therapy agent. This research sheds light on the discrepancies in Osimertinib treatment’s effects and provides valuable insights into its influence on cancer cell behavior.

#### Embryonic-like Torpor/Diapause

As a concept parallels the survival strategies observed in nature, where organisms enter a state of reduced activity to endure difficult environments,^62^ some studies^13,51^ propose that akin to organisms adopting a conservative state of being to survive in hostile environments, such as torpor, estivation, and diapause, cancer employs a similar conservative survival strategy known as the DTP cells in response to the adverse conditions caused by chemotherapy and targeted therapy.^63^

Rehman et al. conducted a study^13^ to examine the factors influencing the DTP cells’ transition in a model of colorectal cancer. They employed various techniques, including second-generation sequencing and mathematical modeling, in their research. The results revealed that all cancer cells have an equal ability to transfer to the DTP cells and that they adopt a state parallel to embryonic stasis to withstand the effects of chemotherapy. Similar to embryonic cells, DTP cells can revert to a cancer state after the cessation of stress. When the pressure is removed, DTP cells can exit this state and develop into cancer again.^64^

#### Stem-like phenotype

Stem-like phenotype may also be a commonly shared special biological trait of DTP cells. The concept of stemness is frequently discussed with cancer stem cells, which form a subset of cancer cells responsible for sustaining malignant growth within a tumor. These cells are typically characterized by self-renewal and pluripotency, demonstrating multidirectional transformation. They can be identified through specific markers such as CD133, CD44, CD166, CD24, and ALDH. The concept of stemness has been particularly instrumental in distinguishing the direction of differentiation and the degree of malignancy across a wide spectrum of tumors.^65,66^

In a study conducted by Arasada RR et al.,^67^ residual cells with a high expression of potential cancer stem cell markers, including ALDH, were observed. These cells, which can be referred to as the DTP cells, were obtained by treating EGFR-mutated lung cancer cells with erlotinib. The researchers found that these residual cells possessed the ability to form spheres with clonal expansion potential and the condition of temporary stasis. This suggests that these cells may play a crucial role in cancer maintenance and resistance to treatment. Similarly, various other cancer types express numerous stem cell markers in DTP cells, including CD133 and CD24 in NSCLC,^53,68^ SOX2, OLIG2, and NFIA in glioblastoma,^69^ and JARID1B and CD271 in melanoma.^70^ In addition, colon,^71^ breast,^72,73^ prostate,^74^ and ovarian^75^ cancer also harbor “dormant cells” exhibiting a stemness phenotype. Cancer cells often exhibit stemness as an adaptive response to environmental stimuli, particularly chemotherapy, leading to the differentiation of cancer cells into various clonal subpopulations.^76,77^ This process is akin to the concept of cellular plasticity, which involves the acquisition of specific molecular traits through subcellular self-renewal, influenced by various intracellular pathways impacting epigenetic and transcriptional regulation. Consequently, these pathways ultimately dictate the resulting cellular behavior.^32,78^ In the case of melanoma cells known as the DTP cells, a transcriptional state associated with the stemness of neural crest cells emerges in response to targeted therapy,^55^ indicative of cellular plasticity. This phenomenon can be described as either melanoma DTP cell stemness or cellular plasticity. Consequently, several articles often draw connections between cancer cell stemness and cellular plasticity.

#### Reversibility of transformation

The reversibility of the DTP cells^79^ is a critical property, as evidenced by the observed cell-cycle inhibition and embryonic-like stasis, rather than a one-way state that cannot be withdrawn. When cancer cells enter this state, they exhibit near-stagnant behavior, allowing DTP cells to retain the potential to become active again and resume proliferation.^7,80^ Consequently, this resurgence is translated of drug-resistant properties and ultimately culminates in drug resistance and cancer recurrence. These observations underscore the significance of the DTP cells’ reversibility in driving the progression toward drug resistance and cancer metastasis.

In a recent study on the DTP cells in breast cancer, Chang et al.^81^ induced breast cancer cells to enter the DTP through the use of TKI inhibitors such as lapatinib and tucatinib. After induction, it was observed that HER2 TKI DTP cells exhibited signs of re-growth upon the cessation of TKI inhibitors. Furthermore, cancer cells exposed to diluted concentrations of TKI inhibitors showed some degree of proliferation and multiplication. Additionally, this subset of cancer cells that had transitioned back to the proliferative and reproductive state were capable of re-entering the DTP, displaying corresponding phenotypic characteristics upon treatment with the appropriate level of TKI inhibitors. These findings conclusively demonstrate that the DTP cells have reversible conditions with the ability to self-regulate in response to environmental changes. Furthermore, the reversible manifestation is not solely present in the DTP cells of breast cancer, but also in ovarian,^82^ colon,^83^ and lung^84^ cancer. Several sources have reported similar response patterns to retreatment after the cessation of treatment.^85^ These findings are discussed in Cara et al.‘s^86^ research, which suggests that a “seemingly drug-resistant but not drug-resistant cancer” may reflect the DTP cells’ reversibility.^87^

The existence of reversibility within DTP cells allows for a small number of cancer cells to persist in the body for an extended period through flexible transformation of the DTP cells, complementing the stemness or plasticity of these cells. This attribute poses significant challenges to eradicating these cells through multiple comprehensive treatments, contributing to the potential for cancer recurrence. However, due to the reversible nature of the DTP cells, investigating the mechanism of this state and developing therapeutic approaches targeting it can facilitate progress in treating drug-resistant cancer and preventing cancer recurrence.

Existing researchers have incrementally developed an initial understanding of DTP cells by integrating observed features. The correlation among these features suggests that DTP cell formation is a holistic and unified process. The characteristics of slow-cell-cycle and reduced proliferation are often expressed in diapause cancer cells.^51,88^ The static stage and recovery stage of the diapause state of these special cancer cells also demonstrate the rationality of their reversible existence.^89–92^ Furthermore, the characteristics of the slow-cell-cycle provide a reserve basis for the stemness or plasticity of DTP cells in the multidirectional differentiation preparation stage.^93–95^

### DTP formation as an inert specialization in response to anticancer therapy

After analyzing and summarizing these special features, it is apparent that DTP cells exhibit a complex mechanism of formation characterized by inert specialization in response to therapeutic stimuli. This process involves cell-cycle restriction, diapause, stemness, and reversibility, which are shared characteristics with certain other specialized cancer cells. However, it is important to note that the essence of DTP cells is not entirely consistent with that of other cancer cells.^96^ The key distinction between DTP and cancer stem cells or dormant cancer cells is their heightened resistance to external drug stimulation. Additionally, their characteristics (Table 1), including origin, phase of existence, proliferative capacity, and tendency for differentiation, vary.

**Table 1:** Related characteristics among DTP cells, cancer stem cells, and dormant tumor cells

	DTP cells	Cancer stem cells	Dormant tumor cells
Origin	Drug-induced formation	Original mutation unit of cancer cells	Metastatic or residual part transformation
Exist phase	Anticancer drug treatment period	Early stages of tumor development and differentiation	Early metastasis or Minimal Residual Disease to resist the immune phase
Proliferation	Low But proliferation restores after the drug withdrawn	High Own ability to unlimited multiply and self-renewal tendency	Extremely Low Hardly proliferate when they are insufficient to counter immunity
Stemness	Some certain degree of stemness and differentiation exists to response to drug stimulation	High degree of stemness and high differentiation tendency, main significance is tumor heterogeneity	Low degree of stemness and almost exclusively change to ordinary tumor cells
Stress memory	Possible some degree of memory ability	No relevant memory ability	No memory ability, even no responding to stress
Resistance	Clearly have resistance	Occasionally have mutagenic resistance	May have limited resistance
Resistance spectrum	Extensive drug resistance	Mono-drug or multi-drug resistance	Usually manifests as silence in response to stimuli, including drugs
Resistance mechanisms	Mechanisms of gene mutation and non-genetic regulation	Absence of certain drug sensitive targets resulting from mutations	The self-silence, low metabolism, and survival logic with little interaction
Markers	Genetic markers: Mex3a,^15^ APOBEC3^304^	Stem associated surface antigen markers: CD133,^305^ ALDH,^306^ CD90,^307^ CD44,^308^ EpCAM^309^	Dormancy related markers: DEC2/Sharp1,^310^ p27,^311^ p21,^312^ NR2F1^313^
	Non-genetic specific markers: ALDH,^37^ PINK-1,^38^ KDM5A^4^		

Compared with completely formed drug-resistant cancer cells, DTP cells exhibit characteristics of a slow-cell-cycle and are somewhat inactive; however, they possess little inherent ability of drug-resistant cancer cells to absorb nutrients and persist in proliferating. DTP cells enter a diapause state, leading to the emergence of more generalized and broad-spectrum drug resistance phenomena in terms of drug response, rather than the specific drug resistance reactions seen in drug resistant cancer cells.^97^ Moreover, there is a notable difference in the reversible nature of drug resistance between the two cell types. The majority of drug-resistant cancer cells undergo genetic changes^98^ that prevent them from reverting to their original non-resistant state, which contrasts with the potential reversibility observed in DTP cells.

For another type of cancer stem cells that is often mentioned to be similar to DTP, the drug resistance of DTP is a significant feature that cancer stem cells cannot have.^99–102^ While both types of cells share similarities in terms of stemness, plasticity, and some surface antigen CD molecules,^103,104^ cancer stem cells primarily function as a reserve subgroup for multidirectional differentiation within cancer cells.^105,106^ DTP is more of a resister of cancer cells stimulated by external drugs. And the plastic ability of DTP can be compared to an instinctive survival mechanism.^107^ Moreover, the extremely high proliferative activity^108–110^ of cancer stem cells contrasts with the slow-cell-cycle of DTP cells,^105^ underscoring their distinct natures. In this sense, many of what were once called cancer stem cells can be defined as DTP cells that live for survival rather than stem cells that exist for differentiation and proliferation.

After differentiating DTP cells from other types of cancer cells with similar properties and analyzing their characteristics before and after formation, it becomes evident that DTP formation serves as an inert specialization response to therapy, transitioning from a state of inactivity to activity following the conclusion of therapeutic stimulation. This phenomenon poses challenges in fully elucidating the mechanisms behind cancer recurrence and drug resistance, as research neglects the subtle changes in cancer cells during drug treatment. This knowledge gap may underlie the persistent difficulties encountered in addressing drug resistance in cancer. However, by enhancing the understanding of DTP, it becomes conceivable to overcome the obstacles hindering the effective treatment of drug resistance in cancer.

### The observed memory effects after DTP cells formation

Additionally, DTP cells exhibit a special feature that cannot be generalized to biological characteristics, but also makes it different from other special cancer cells: several studies^60,70^ have revealed a memory effect following the creation and reversible restoration of DTP, implying a potentially significant role in the development of genetic, stable, and irreversible drug resistance. Specifically, DTP cells emerging during anti-EGFR therapy in lung cancer demonstrate a remarkable level of resilience that permits the cells to have a highly stable chance of sustaining a response, enabled by the phosphorylation of insulin receptor substrate 1 (IRS1).^111^ The phosphorylated state bestows various long-term memories upon the modified DTP cells, allowing them to present heritable components when the relevant drug is reintroduced following a treatment interruption. The preservation of epigenetic changes in DTP cells as a heritable memory upon reverting to a drug-sensitive state creates a pathway for the evolution of drug-resistant entities. The process raises the possibility of increased persistence in cells upon drug rechallenge, consequently escalating the frequency of DTP formation and potentially enhancing rates of drug resistance like EGFR-resistant lung cancer cases. While the ultimate impact on drug resistance evolution about DTP cells requires further investigation, the observed memory phenomenon and the terrible likelihood of DTP cells enrichment warrant continued research and validation.

## Being explored DTP: Possible mechanisms underlying DTP cells formation and drug resistance

The fact that the DTP cells are potentially reversible and memorable accentuates their pivotal importance as an exploratory subject for investigators. Understanding the biological traits of the DTP condition may not fully elucidate its capacity to withstand the lethal impact of cancerous cells linked to treatment. Therefore, comprehending how the DTP cells arise mechanistically is crucial for the ultimate goal of reversing the DTP cells for therapeutic purposes. The process of forming the DTP cells remains incompletely understood and can vary between cancer cells of distinct cancer, making the study of this process more challenging. Consequently, the mechanisms of DTP cells’ formation are both specific^112^ and universal,^13^ highlighting the complex nature of this phenomenon.

The mechanism of DTP formation involves important considerations that need to be examined: i) At a vertical level, there are distinctive characteristics of DTP cells that make them easily distinguishable from pre-forming parental cancer cells and post-resuscitation filial cancer cells and have unique advantages in responding to treatment and demonstrating drug sensitivity.^113^ ii) At a horizontal level, there are prevalent parallel features that are common to various cancer DTP cells, indicating a commonality across the DTP transformation process of different cancer types.^16,61^ Consideration of the mechanism of state formation in conjunction with these two aspects will result in a more generalizable and reliable understanding.

**Fig. 4:**
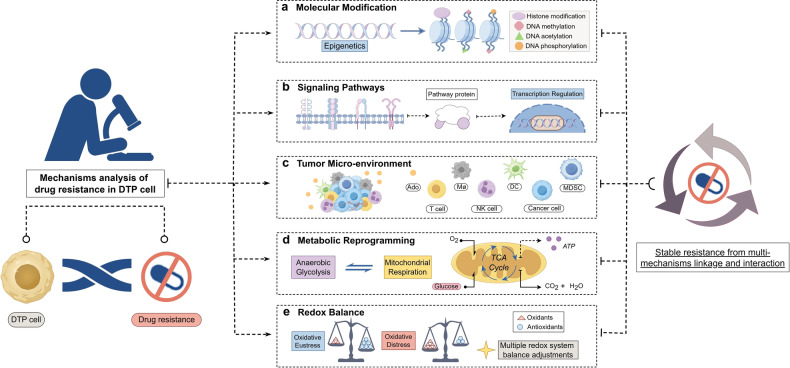
Multi-mechanisms analysis of DTP resistance. Molecular Modification (**a**): DTP cells exhibit a significant number of molecular modifications as compared to parental tumor cells, encompassing DNA methylation, acetylation, phosphorylation, and histone modification. Signaling Pathways (**b**): Signaling plays a crucial role in cancer cells, and altered pathways in DTP cells contribute to transcription regulation, DTP formation, and drug resistance progress. Tumor Micro-environment (**c**): TME surrounding the DTP cells changes to accommodate both drug action and the environment’s alterations. Metabolic Reprogramming (**d**): DTP cells rely more on mitochondrial respiration than most cancer cells that survive using glycolysis, for their energy needs. Redox Balance (**e**): The robust redox system within DTP cells enables them to withstand the balance between oxidative eustress and distress. These multi-mechanisms interact with each other and together provide the stable basis for drug resistance in DTP cells. (Drawn by figdraw)

This review synthesizes the findings of prior long-term and continuous investigations, outlining the primary mechanisms that give rise to DTP cells (Fig. 4): molecular modification regulation, signaling pathways and transcription regulation, tumor micro-environmental transformation regulation, metabolic shifts regulation, and redox regulation (which is often overlooked but holds significant importance). These mechanisms do not exist in isolation and are not operating under either/or single mechanism conditions.^114–117^ In most cases, multiple mechanisms work together when cancer cells enter DTP, with one mechanism augmenting another,^118,119^ rendering DTP cells resistance persistent and challenging to treat and eliminate. In the ensuing segment, we will analyze each mechanism’s traits and interactions point by point.

### Regulation of molecular modification and epigenetic reprogramming

The frequent occurrence of molecular modification in cancer involves alterations in DNA or RNA promoter regions, histone acetylation, methylation, or variations in repetitive element expression.^120,121^ Cancer cells exploit these epigenetic modifications to induce their heterogeneity and develop drug resistance.^122–126^ Consequently, DTP entities can likely employ analogous non-genetic molecular modifications to initiate epigenetic reprogramming, thereby leading to the emergence of their drug resistance. This shared mechanism is anticipated to play a significant role in drug resistance.

Sharma et al.^32^ found that the epigenetic regulator histone H3K4 demethylase KDM5A is activated during the formation of the DTP cells in EGFR mutant lung cancer cells. Concurrently, Buenrostro et al.^127^ also documented epigenetic reprogramming resulting from such molecular modifications. Additionally, the activation of KDM5A was shown to be crucial in inhibiting cell-cycle progression.^128–130^ A well-established review^131^ highlights the significant involvement of KDM5A in oncology, closely associating it with cancer growth, differentiation, drug resistance, invasion, and metastasis. Criscione et al.‘s^61^ study investigated the use of Osimertinib for the treatment of NSCLC and its role in inducing the generation of DTP cells. The study revealed that the DTP cells displayed a distinct gene expression profile compared to the cancer cells eliminated by direct Osimertinib treatment. Specifically, the DTP cells exhibited up-regulation of YAP1 and TEAD genes, and increased susceptibility to inhibition of BRD4, AURKB, and TEAD targets. Moreover, the study found that the expression of MEK-activated genes, including MAPK13, EPCAM,^132^ ETV4,^133^ DUSP4,^134,135^ and PHLDA1,^136–138^ was down-regulated in the DTP cells. Conversely, MEK compensatory resistance genes, such as IGFBP3,^139,140^ MAP2,^141^ and SERPINE1,^142^ were upregulated and contributed to resistance to anticancer therapy with EGFR TKI in the DTP cells.^143^ Noronha et al.^144^ observed the up-regulation of Growth Arrest-specific Protein 6 (GAS6) in residual cells of anti-EGFR treated lung cancer, leading to the subsequent formation of DTP cells. They also found a significant association between the expression of AXL, the receptor for GAS6, and the formation of the DTP cells and drug resistance. Moreover, overexpression of AXL has been demonstrated to enhance DTP cells’ activity and promote the emergence of the common EGFR mutation T790M in drug resistant lung cancer.^145–147^ The mechanism underlying this effect may involve AXL-induced activation of low-fidelity DNA polymerase and RAD18 through neddylation promotion. Additionally, AXL activation of MYC may lead to an increase in purine synthesis due to pyrimidine imbalance,^148^ further contributing to drug resistance.^148,149^ Additionally, a detailed analysis^150^ of receptor tyrosine kinase (RTK) activation using phosphorylated RTK arrays indicated that exposure of HCC4006 cells to erlotinib leads to the induction of the DTP cells, activating HER family and MET^151,152^ targets. Based on the previous statements, it is clear that epigenetic reprogramming through molecular modification plays a crucial role in the development of DTP cells and the subsequent emergence of drug resistance.

### Regulation of signaling pathways and transcription

Cancer cells are characterized by the presence of numerous specific signaling pathways and dysregulated transcription factors that play a critical role in driving their abnormal proliferation and growth.^153–155^ Key therapeutic approaches involve the use of targeted drugs aimed at these specific signaling pathways, which have been identified as crucial targets in anticancer therapy.^156,157^ Notably, the EGFR signaling pathway and its downstream JAK/STAT,^158^ PI3K/AKT,^159,160^, and MAPK^161,162^ signaling pathways^163^ are among the main targets identified. These pathways play multiple roles in tumor development and have been implicated in drug resistance mechanisms in certain cancer cells.^164–167^ Additionally, the regulation of molecular modification, previously discussed, directly impacts these signaling pathways and transcription. Therefore, researchers are investigating the potential mechanistic link between signaling pathways and transcriptional changes in the DTP cells by scrutinizing and comparing the signaling pathways and transcription factors of typical cancer cells with those of DTP cells, thereby elucidating the differences between the two.

The molecular modification of the DTP cells involves several key mechanisms: Firstly, the upregulation of AXL expression can be induced by anti-EGFR treatment, which disables the negative feedback loop of SPRY4.^168^ Consequently, this activation inhibits the cancer cell-cycle through the signaling pathway axis via AXL and its ligand GAS6.^169^ Secondly, the upregulation of the YAP and TEAD genes is related to the action of the Hippo pathway.^61,170^ YAP, TEAD, and their complexes function as transcription factors that contribute to the formation of the DTP cells and impact drug sensitivity.^156,170^ Furthermore, studies^171^ have indicated that YAP/TEAD may influence the EMT-associated transcription factor SLUG^172,173^ and impede the apoptosis of cancer cells caused by therapeutic drugs. These examples illustrate how molecular modifications affect the signaling pathway shift, playing a significant role in the formation of the DTP cells.

After treatment with the BRAF inhibitor Vemurafenib in the BRAF (V600E) melanoma model, residual cancer cells have been observed to exhibit compensatory up-regulation of EGFR targets and other pathways associated with overcoming therapeutic drug-induced inhibition of the MAPK pathway.^174^ It is noteworthy that targeting multiple sites along the EGFR signaling pathway and its downstream pathways, through the combined use of a BRAF inhibitor and an EGFR inhibitor, has shown greater efficacy in treating drug-resistant BRAF (V600E) colon cancer patients.^175,176^ This observation suggests that alterations in signaling pathways, especially those related to cell proliferation, may have a compensatory effect and participate in the emergence of the DTP cells and the mechanisms that confer drug resistance.

The Wnt/β-catenin signaling pathway is a widely studied association with DTP cells. According to Nie et al.,^177^ transcriptomics results suggest that YAP can mediate the metabolism of Ach by increasing the expression of the biosynthesis-limiting enzyme choline acetyltransferase ChAT in response to EGFR TKI. This results in the specific accumulation of Ach in DTP cells, ultimately activating the Wnt/β-catenin pathway through Ach Muscarinic Receptor 3 (M3R)^178,179^ and stimulating β-catenin signaling through the Notch3 pathway.^180^ Notably, the mutant aberrant activation of the Wnt pathway is a major factor driving most types of tissue stem cells, contributing to the development^181^ of the DTP cells and the stemness^182–184^ of DTP cells.

The formation of the DTP cells involves the identification of several other signaling pathways. Osimertinib treatment has been found to upregulate the Notch pathway in DTP cells,^185^ and this pathway significantly interacts with the EGFR pathway. Moreover, the IGF-1R pathway and its associated Akt pathway, linked to KDM5A and H3K4 targets, are also modulated in DTP cells.^186^ Additionally, the activation of the transcription factor FOXA1 has been observed in EGFR mutant NSCLC treated with Osimertinib, resulting in the upregulated expression of IGF-1R and induction of the DTP cells.^187^

**Fig. 5:**
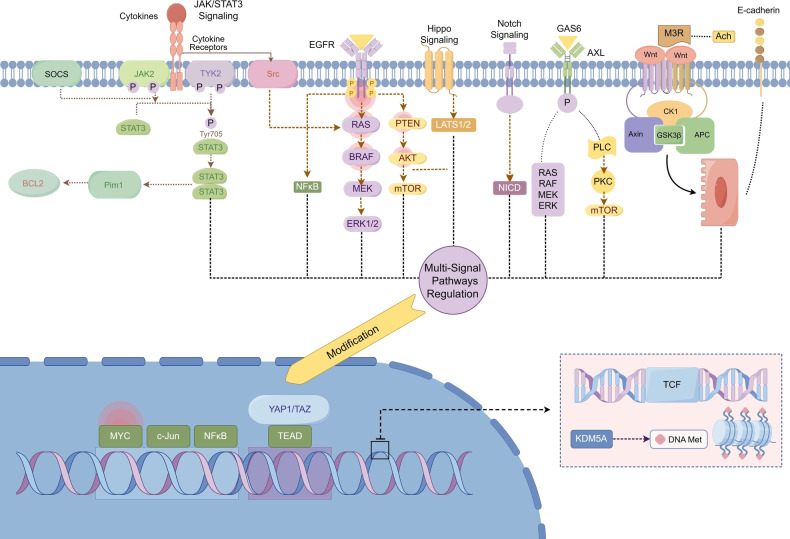
As shown above, DTP cells undergo numerous modifications to genes and signaling pathways. Important modifications include KDM5A-mediated DNA methylation modification, increased expression of GAS6/AXL, adjustments to EGFR, JAK/STAT3 and their downstream pathway, activation of the Hippo pathway and its YAP/TEAD branch, the Notch pathway, the Wnt/β-catenin pathway, and modulation of E-cadherin. Ultimately, the drug resistance of DTP cells has been affected. (Drawn by figdraw)

The association between certain pathway changes and resistance to DTP cells is established, while others are still under investigation. Nevertheless, numerous signaling pathways are undoubtedly altered during the formation of the DTP cells (Fig. 5).

### Regulation of tumor micro-environmental transformation

The tumor micro-environment, located at the site of the tumor, comprises non-cancerous cells such as stroma and immune cells, and it plays a crucial role in tumorigenesis, progression, metastasis, and treatment response.^188–190^ Influenced by tumor factors, this micro-environment can lead to drug resistance in several ways.^191–193^ During the transition of cancer cells to DTP cells, the composition and contents of the micro-environment undergo a transformation as cancer cells and cancer associated fibroblasts (CAFs)^194–196^ directly or indirectly influence it, to adapt to the corresponding changes and demands.

The micro-environmental transformation includes epithelial-mesenchymal transition (EMT), epithelial cell development, vesicle-mediated transport, drug metabolism, and cholesterol homeostasis.^42^ Studies have indicated the association of this transformation with molecular modifications^197,198^ and alterations in signaling pathways^199,200^ during the development of the DTP cells. Furthermore, changes in the micro-environment were evident in environments associated with extracellular matrix (ECM) secretion, EMT, the TGF-β/SMAD signaling pathway, and other processes.^61^ Also noted is a micro-environmental state of high mesenchymal state.^201^ The modification of the mesenchymal state, induced by the EMT regulator with the lipogenic factor ZEB1,^202,203^ leads to an elevation in enzymes responsible for polyunsaturated fatty acid synthesis. This increase in enzyme activity results in a deficiency of substrates for lipid peroxidation by lipoxygenase, subsequently reducing the presence of lipid peroxides and lowering the risk associated with drug therapy. Therefore, the transformation in treatment is critically linked to the altered mesenchymal state driven by the EMT regulator and ZEB1, which effectively modifies the cellular environment to mitigate lipid peroxidation and the drug’s efficient effects.

The crucial role of the micro-environment associated with DTP in forming drug resistance has been highlighted by a study conducted by Suda et al. ^150^. In this study, a TGF-β-mimicking EMT state^204^ was utilized to culture HCC4006 cells, resulting in the generation of erlotinib-resistant DTP cells, denoted as HCC4006ER. The findings of the study revealed that the TGF-β cultured cells exhibited resistance to erlotinib, similar to the erlotinib-resistant DTP cells. The subsequent analysis^150^ through immunoblotting revealed that both HCC4006ER and DTP cells showed SMAD2 phosphorylation^205^ and down-regulation of E-calmodulin.^206^ Moreover, it was observed that erlotinib sensitivity in HCC4006 cells, which were cultured with TGF-β, was restored upon withdrawal of TGF-β. The formation of DTP cells and the associated changes to the tumor micro-environment raise questions about their causal relationship. It is uncertain whether DTP cells initiate changes to the tumor micro-environment or if the micro-environmental transformation precedes the development of DTP cells. While DTP cells may not directly instigate changes in the tumor micro-environment, the tumor micro-environment plays a crucial role in DTP formation and its acquisition of drug resistance. Therefore, attributing them to the mechanism of DTP formation is a justifiable approach.

### Regulation of metabolic reprogramming

The alteration of energy metabolism stands out as a hallmark of cancer cells, characterized by a key marker named the Warburg effect.^207^ According to current knowledge, cancer cells favor the utilization of anaerobic glycolysis as their primary pathway for energy acquisition,^208,209^ involving the conversion of glucose to pyruvate and subsequently to lactate. On the other hand, DTP cells display a preference for utilizing mitochondrial respiration and the electron transport chain to acquire energy.^32^ This distinction in energy acquisition pathways underscores the metabolic differences between cancer cells and those DTP cells, indicating the potential for targeted therapeutic strategies based on metabolic alterations in cancer cells. Raha et al.^41^ proposed that elevated ALDH levels may be indicative of cancer cell conversion into DTP cells. To investigate this claim, the researchers conducted a study comparing homozygous cancer cells with different levels of ALDH expression. They measured the oxygen consumption rate (OCR), an indicator of mitochondrial electron transport chain (ETC) activity. Their findings revealed that cells with high ALDH expression displayed an increased basal OCR, along with elevated mitochondrial respiration and ROS production. Subsequent analysis and other research^210^ demonstrated that cells exhibiting high OCR and increased ROS production were capable of expressing higher levels of ALDH1A1. This suggests that DTP cells may rely on oxygen and mitochondria for survival.

Currently, the reliance on mitochondrial respiration has been demonstrated in drug-resistant cancer cells and DTP cells from multiple cancer sources.^211–215^ In BRAF-mutated melanomas, blocking the mitochondrial respiratory chain of slow-cell-cycle JARID1B(high) cells, now referred to as DTP cells, has been proven effective in inhibiting their drug resistant behavior.^78^ Furthermore, in the field of triple-negative breast cancer treatment research,^49^ Phase 1 clinical development is underway to investigate the efficacy of an oxidative phosphorylation inhibitor. Researchers have reported that this inhibitor^49^ can target cancer cells in a reversible drug-resistant state and hinder the effects of residual tumor regeneration, indicating its potential as a promising treatment strategy.

The development of DTP cells is partly characterized by a metabolic shift, leading to an increased reliance on oxidative phosphorylation for energy, as observed in the above studies.^41,49^ Consequently, there is a concurrent rise in ROS levels within the cells. This elevation in ROS triggers a natural mechanism of redox regulation, which serves to counteract not only ROS but also other metabolites in the cellular environment.

### Regulation of integrated complex redox systems

Cells have developed intricate detoxification mechanisms, including the production of molecules with strong reducing properties, such as cysteine and glutathione, in response to the accumulation of toxic by-products caused by the production of oxidative metabolites in cellular metabolism and other cellular activities.^216,217^ The detoxification mechanisms also involve the elimination of toxic oxidative metabolites, including peroxides and lipid peroxides,^218–220^ that can cause cellular damage. This signifies the adaptive response of cells in mitigating the potential harm caused by the accumulation of these toxic by-products.

**Fig. 6:**
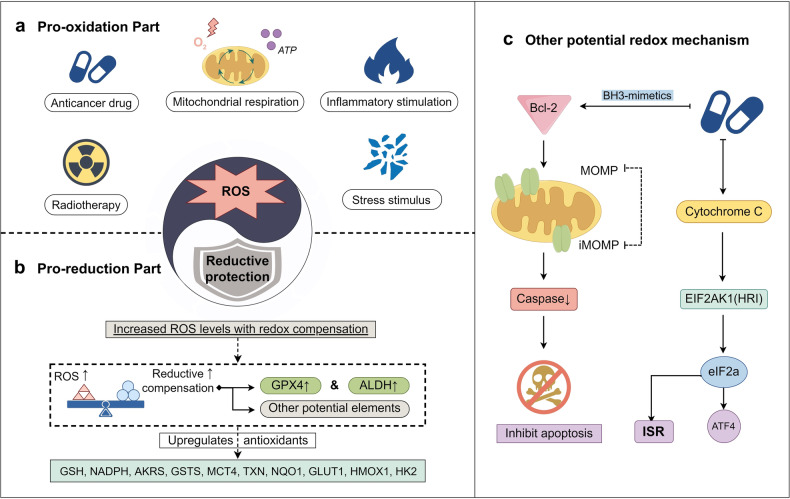
Redox regulation in DTP cells. The Redox System Balance (**a**/**b**) is influenced by mitochondrial respiratory metabolism, anticancer therapeutic drug effects, radiotherapy, inflammatory stimulation, and external oxidative stress. These factors elevate the ROS level of DTP cells (Pro-Oxidation Part). Then DTP meets these oxidative stimuli by multiple reductive protection mechanisms with redox compensation like GSH/NADPH/HK2 through enhancing the expression of GPX4 and ALDH, and other potential related elements (Pro-Reduction Part). The balance between the two ensures the redox stability of DTP cells. Other potential redox mechanism (**c**): external drug stimuli, particularly BH3-mimetics can trigger an incomplete mitochondrial outer membrane permeabilization (iMOMP) in DTP cells by acting on Bcl-2 family anti-apoptotic proteins (including BAX, BAK, BOK, etc.) and reducing caspase activation. Drug treatment protects DTP cells from apoptosis by inhibiting membrane permeabilization and caspase activation. Additionally, a protective mechanism against oxidative stress in DTP is stimulated by the drug, which acts on the cytochromeC-HRI-eIF2a-ISR/ATF4 axis. (Drawn by figdraw)

Cancer cells, although they are a less harmonious part of the organism, are subject to the same basic redox rules.^221,222^ These cells have their redox mechanisms to protect themselves from the oxidative by-products of their metabolism.^223^ However, the protective capacity of these mechanisms is limited, and external factors such as antineoplastic drugs disrupt the balance^224^ between the redox protection mechanisms maintained by normal cancer cells and the oxidatively-derived damage caused by external factors.^225–227^ As a result of this disruption, normal cancer cells become damaged and transformed (Fig. 6). To address the redox imbalance that occurs when cancer cells transition to the DTP cells, it is important to adjust and reconstruct the redox regulatory mechanisms and effectively respond to the negative effects of drug therapy.

Reactive oxygen species (ROS) are indispensable for maintaining the normal redox system.^228,229^ By inducing oxidative stress in cells, ROS can disrupt the cell’s antioxidant system established for homeostasis, ultimately leading to cell death. Notably, cancer cells are not immune to the effects of ROS, and some drugs harness this phenomenon to selectively eliminate cancer cells for therapeutic purposes. The formation of DTP cells not only showcases redox-regulated mechanisms but also reveals their resistance to pro-ROS drugs. This resistance is supported by the metabolic changes mentioned earlier, in addition to the enhanced mitochondrial functioning of DTP cells, which involves their ability to acquire energy through oxidative phosphorylation and other mechanisms associated with elevated levels of ROS.^230^ Zhang et al.^33^ discovered a higher concentration of GPX4 and ferrous ions in DTP cells obtained from a colorectal cancer model treated with 5-FU. GPX4, also known as glutathione peroxidase 4, can utilize GSH to directly reduce phospholipid peroxide, thereby protecting cells from oxidative stress induced by chemotherapy.^231–233^ Other studies^234,235^ have also reported the protective role of GPX4 in DTP cells against oxidative stress. Furthermore, ferrous ions, as a component of iron transport, play a role in the redox mechanism, which aligns with the association between total iron content, labile iron pool (LIP), and high CD44 expression^57^ in DTP cells. These findings suggest that the transport of iron involved in the redox mechanism may contribute to drug resistance. Subsequent studies involved the knockout of the GPX4-related gene in DTP cells, which revealed that GPX4 KO led to significant sensitivity to lipid peroxidation in the primary and mild sensitivity to ordinary oxidative stress in the secondary. Moreover, GPX4 KO DTP cells exhibited oxidative stress damage that could not be rescued by the antioxidants Ebselen and EUK134 but could be rescued by the lipophilic antioxidant ferrostatin-1.^8^ Collectively, these findings point to the reliance of DTP cells on GPX4.^32,41^ Loss of GPX4 function in vitro resulted in selective DTP cells death due to iron, suggesting the potential to prevent cancer recurrence.^236,237^ Furthermore, a comparison between DTP cells and their parental common cancer cells revealed that DTP cells display heightened sensitivity to GPX4 inhibition, increased levels of lipid ROS, elevated expression of reduced GSH-specific γ-glutamyl transferase 1 (CHAC1), and reduced expression of glutamate-cysteine ligase catalytic subunit (GCLC), indicating the necessity of GPX4 for DTP cells.^238^ Furthermore, in the maintenance of the DTP cells, the expression of ALDH (aldehyde dehydrogenase) is considered essential. Cancer cells with high ALDH expression have been found to exhibit greater resistance than those with low expression. Raha et al.^41^ conducted a study on the gene expression changes in DTP cells derived from the MET-induced gastric cancer cell line MKN-45, before and after treatment with the MET kinase inhibitor Crizotinib. The study revealed that the drug-resistant subpopulation exhibited increased ALDH1A1 expression. Although multiple ALDH isozymes exist, ALDH1A1 is particularly significant. However, the researchers found that knocking down ALDH1A1 alone did not hinder DTP formation. Instead, they observed increased expression of other ALDH isozymes, indicating potential redundancy within the ALDH family in response to drug-induced upregulation. ALDH is involved in the redox mechanism and facilitates the formation of DTP cells. Its related proteins play a role in catalyzing the oxidation of metabolically produced aldehydes, thereby controlling their cell-damaging effects. This scavenging activity may contribute to DTP formation as these enzymes can detoxify cancer cells from toxic aldehydes produced by certain chemotherapeutic drugs,^239^ contingent upon the cellular redox state. Additionally, the presence of ALDH may protect cells against the toxic potential of ROS.^240,241^ Disrupting ALDH activity with drugs could result in the accumulation of toxic levels of ROS, leading to DNA damage and apoptosis in a subset of DTP cells.^41^ Kalkavan et al.^238^ discussed a study involving sublethal mitochondrial outer membrane permeabilization (MOMP) and the cytochrome C related redox regulated transformation of DTP cells. The research started with an investigation of commonly used drugs that target the apoptosis-intrinsic pathway for treating DTP cells. It was found that BH3-mimetics, designed to act on Bcl-2 family effector proteins (specifically BAX, BAK, and BOK, which are responsible for mitochondrial outer membrane permeabilization MOMP-induced apoptosis^242,243^), have a BH3-like impact on apoptosis induced cell death. The binding of BH3-only proteins to anti-apoptotic proteins, either via their BH3 domains or by directly binding with BAX and BAK to form MOMP, results in incomplete mitochondrial outer membrane permeabilization (iMOMP).^244^ This iMOMP event induces minimal caspase activation, which in turn promotes the protection of cell survival and contributes to the formation of DTP cells.^245,246^ In addition, therapeutic agents induce upstream stress that affects the cytochrome C in the cytoplasm of cancer cells, leading to the activation of the heme-regulated inhibitor EIF2AK1 kinase (HRI, which also contains EIF2AK2/PKR, EIF2AK3/PERK, EIF2AK4/GCN2). This activation leads to the phosphorylation of the translation initiation factor eIF2a, resulting in translational reprogramming and the inhibition of global cap-dependent translation. Thereafter, it participates in the integrative stress response (ISR) and activates the transcription factor ATF4, which initiates the corresponding gene expression program. This process ultimately increases nutrient uptake and autophagy in the short term, suppressing oxidative stress to promote survival.^247,248^

Considering these redox regulated adaptive changes, it is evident that the fundamental link between the DTP cells and cellular homeostasis is facilitated. These regulatory systems enable cancer cells to enhance their adaptive capacity when confronted with oxidative stress damage induced by treatment, thereby playing a pivotal role in the formation of DTP.

### Linkage and interaction between mechanisms

The close connection between molecular modification, signaling pathway regulation, tumor micro-environment regulation, metabolic transformation, and redox homeostasis synergistically and collectively supports DTP cells in resisting the cytotoxic effects of drugs.

Both molecular modifications and signaling pathways are intertwined in a symbiotic relationship.^249^ Molecular alterations instigate changes within their corresponding signaling pathways, setting off a chain reaction of adaptations throughout the cellular structure. The consequential cellular transformations and protein responses resulting from these interactions play a significant role in shaping the tumor micro-environment. For instance, the involvement of the type 3 muscarinic acetylcholine receptor (M3R) in signaling pathway regulation is crucial for the phosphorylation of VE-cadherin and β-catenin tyrosine,^250^ thereby influencing the presence and function of cadherin and other factors that regulate the tumor micro-environment.^251^ These modifications and adjustments have cascading effects on the metabolic system, leading to shifts in energy requirements and alterations in metabolite outcomes. Furthermore, redox regulation has far-reaching mutual effects beyond its direct impact, influencing molecular modification, signaling pathway regulation, and causing micro-environmental shifts that facilitate the transition of regular cancer cells into a slow-cell-cycle state. Puig et al.^252^ showcased the role of oxidative stress-regulated TET enzymes in cancer cells, which directly impact DNA methylation, initiating the transition to a slow-cell-cycle state and promoting the formation of DTP cells. This demonstrates the direct influence of redox regulation on molecular modification and the promotion of a slow-cycling state. The critical role of redox regulation, as a key aspect of metabolism, significantly affects various mechanisms involved in the development of DTP cells.

By optimizing the synergistic combination of different mechanisms, it is possible to mitigate the stress damage induced by cancer treatment, transforming normal cancer cells into DTP cells that are better equipped to withstand such stressors, thus improving survival conditions.

## Worth recognized DTP: Overview of clinical research progress of anti-DTP therapy

After surviving anticancer therapy, DTP cells exploit their inherent biological properties to develop drug resistance, leading to resistance to subsequent therapy or cancer recurrence following treatment. Addressing these hard-to-treat DTP cells through drug intervention represents a breakthrough in anticancer therapy. To achieve this, understanding the formation mechanism of DTP cells and their resistance-related properties provides a basis for subsequent therapeutic management. Accordingly, exploring solutions to reverse the drug-resistant state of DTP cells, leveraging their reversible nature, emerges as a promising avenue for further research.^113^

### Overview of anti-DTP therapy

As researchers deepen their understanding of DTP cells, focusing on how to target the formation of DTP cells and what aspects of the formation mechanism have naturally been a consistent interest. Up to now, the primary strategies of anti-DTP therapy can be summarized in the following parts: (1) Targeting molecular modification to prevent normal cancer cells from converting to DTP cells; (2) Targeting signaling pathways to inhibit the formation of DTP cells; (3) Targeting the tumor micro-environment, where DTP cells exist, to either weaken environmental drug resistance or enhance their sensitivity; (4) Targeting the cyclic hysteresis of DTP cells formation, waking up dormant cancer cells; (5) Targeting the intrinsic conditioned homeostasis of DTP cells necessary for tolerating environmental stresses. Numerous studies^5,8,253^ have underscored the significance of various approaches in tackling DTP cells in cancer treatment. Each approach possesses unique features and practical applications, but they also come with limitations. Despite the implementation of solutions to combat DTP cells, current conditions remain unsuitable for fully eradicating them, as indicated by several studies. However, the positive outcomes of these strategies include a reduction in the presence of DTP cells, reversal of initial drug resistance in some tumors, and decreased possibility of cancer recurrence in the long run.^23^ This highlights the importance of gaining a deeper understanding of these effective drugs in clinical oncology treatment and emphasizes the necessity for further research in this area.^77,110^

**Fig. 7:**
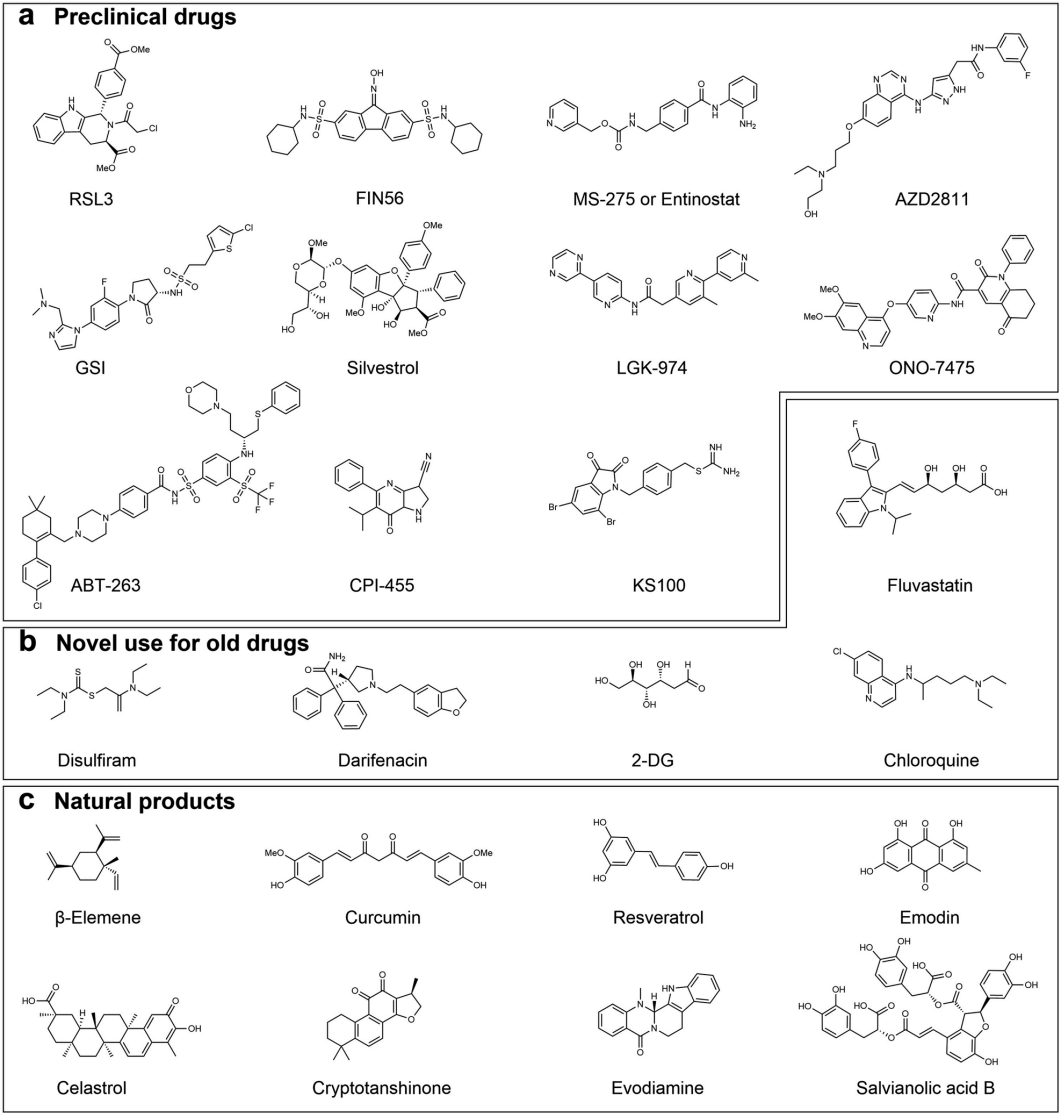
Selected Anti-DTP drugs. **a**: Preclinical drugs; **b**: Novel use for old drugs; **c**: Natural products. (Drawn by chemdraw)

From a clinical perspective, it is essential to consider the overall role of a drug in the human body, which is a complex system, even though studying targeted drugs through their corresponding mechanisms is a suitable direction from a research and exploration standpoint. A narrow focus on the drug’s mechanism of action can lead to over-mechanized thinking and overlook its broader impact. Moreover, solely emphasizing the counter mechanism can lead to blindly pursuing new drug development while overlooking the feasibility and accessibility of medications, thereby neglecting the re-evaluation of other traditional anticancer treatments. This review aims to expand on the subject of anti-DTP treatment to effectively address the above-predisposed bias errors. Specifically, it will examine three different treatment tendencies, namely (a) experimental medicine mode, (b) traditional medicine mode, and (c) traditional Chinese medicine-assisted mode, based on existing traditional treatment, clinical experiments, and Chinese medicine treatment. By considering the scope of current and potential treatment related research, such a classification method review seeks to offer a comprehensive perspective and establish a more robust framework for the selection and implementation of anti-DTP therapy in clinical settings. (Fig. 7, Table 2).

**Table 2:** Summary of three models covering different classes of drugs for DTP cells in cancer

Drug name	Cancer type	Target mechanism	Target point	Side effect	Ref.
Drug Type: Preclinical drugs					
RSL3, FIN56	Colorectal cancer	Redox homeostasis	GPX4	Non-clinical trial	^33^
MS-275	Lung cancer	Molecular modification Tumor micro-environment	HDAC family E-Cadherin	Phase 1/2 Acute toxicity Skin/Eye Irrit.	^150^
AZD2811	Lung cancer	Molecular modification Signaling pathway	Aurora B kinase ATR-CHK1-Aurora B signaling	Phase 1/2 Unknown toxicity	^17^
GSI	Lung cancer	Signaling pathway	NOTCH signaling	Non-clinical trial	^185^
Silvestrol	Glioblastoma & Pancreatic cancer	Signaling pathway Transcription regulation	eIF4A AKT/mTOR, ERK1/2 signaling	Non-clinical trial	^314,315^
LGK-974	Breast & Colorectal cancer	Signaling pathway	Wnt/β-catenin signaling	Phase 1/2 Unknown toxicity	^316–318^
ONO-7475	Acute myeloid leukemia & Lung cancer	Molecular modification	AXL	Phase 1/2 Unknown toxicity	^319–321^
BH3-mimetic (ABT-263, ABT-199)	Prostate & Rectal cancer Lymphoma	Molecular modification Metabolism conversion	Bcl-2	Phase 1/2/3 Unknown toxicity	^254^
CPI-455	Lung & Esophageal cancer	Molecular modification Metabolism conversion	KDM5 Mitochondrial pathway	Non-clinical trial	^284,322,323^
KS100	Melanoma	Molecular modification Redox homeostasis	ALDH family	Non-clinical trial	^324^
Drug Type: Novel use for old drugs					
Fluvastatin	Pancreatic cancer	Tumor micro-environment Redox homeostasis	HMG-CoA GPX4	Hepatotoxicity Reproductive toxicity	^201,325^
Disulfiram	Thyroid & Lung cancer	Molecular modification Redox homeostasis	BMI-1 ALDH family	Hepatotoxicity Reproductive toxicity Skin/Eye/ Respiratory Irrit.	^266,326,327^
Darifenacin	Lung cancer	Signaling pathway	Ach/M3R signaling	Hepatotoxicity Anticholinergic effects	^177^
2-DG	Pancreatic cancer	Metabolism conversion	Hexokinase-2	Acute toxicity	^328,329^
Chloroquine	Liver & Breast cancer	Metabolism conversion Signaling pathway	HGF-MET signaling	Hepatotoxicity Reproductive toxicity Group 3 carcinogen Convulsive seizures Headache	^330,331^
Drug Type: Natural products					
β-Elemene	Ovarian cancer	Molecular modification Metabolism conversion Cycle slowness	Bcl-2/XL, Bax Cyclin A, Cdc2	Acute toxicity	^332–334^
Curcumin	Colorectal cancer	Signaling pathway	Notch/Wnt signaling	Acute toxicity	^335^
Resveratrol	Colorectal cancer	Molecular modification Signaling pathway	Nrf2 NF-κB signaling	Hepatotoxicity Reproductive toxicity	^336^
Emodin	Renal cancer	Redox homeostasis	ROS levels JNK signaling	Electrolyte-unbalance Diarrhea	^271^
Celastrol	Ovarian & Colorectal cancer	Molecular modification Signaling pathway	SIRT STAT signaling	Acute toxicity	^337–339^
Crypto-tanshinone	Melanoma & Prostate cancer	Molecular modification Metabolism conversion Redox homeostasis	Bcl-2 ROS-mitochondrial pathway	Acute toxicity	^340,341^
Evodiamine	Non-small-cell lung cancer & Breast cancer	Signaling pathway	NF-κB, Smad2/3 TGF-β/HGF SOX9-β-catenin signaling	Acute toxicity	^271,342,343^
Salvianolic acid B	Gastric cancer & Colorectal cancer	Signaling pathway Tumor micro-environment Redox homeostasis	AKT/mTOR signaling ROS levels EMT suppression	Unknown toxicity	^344–346^

*2-DG* 2-Deoxy-D-glucose, *GPX4* glutathione peroxidase 4, *HDAC* histone deacetylase, *NOTCH* translocation-associated Notch protein, *eIF4*a eukaryotic translation initiation factor 4A, *AKT* protein kinase B, *mTOR* mechanistic target of rapamycin kinase, *ERK* extracellular regulated protein kinase, *WNT* wingless-type MMTV integration site family, *AXL* AXL receptor tyrosine kinase, *BCL* B cell lymphoma protein, *KDM5* lysine demethylase 5, *HMG-CoA* 3-hydroxy-3-methyl glutaryl coenzyme A reductase, *BMI-1* B-cell-specific Moloney murine leukemia virus insertion site 1, *HGF* Hepatocyte growth factor, *Cdc* cell division cycle, *NRF2* NF-E2-related factor 2, *NF-κB* nuclear factor kappa-B, *JNK* c-Jun N-terminal kinase, *SIRT* Sirtuins protein, *STAT* Signal transducers and activators of transcription, *Smad* drosophila mothers against decapentaplegic protein, *TGF-β* transforming growth factor-β

### Anti-DTP drugs in experimental medicine mode

Participating in clinical trials is often a viable option for patients with relapsed or resistant refractory cancer, warranting the attention of both healthcare providers and patients. The core of this experimental medicine model lies in preclinical drugs, which work by elucidating newly discovered mechanisms and developing new drugs to tackle challenging issues. Among the primary categories of preclinical drugs are those undergoing validation processes or based on theoretical principles to combat drug tolerance and resistance in cancer. An exemplar in this category is the BH3-mimetics,^254^ known for generating numerous Bcl-2 inhibitors and serving as an anti-DTP agent that obstructs molecular modification and metabolic aspects of DTP cells.^255–258^ The widely researched drug ABT-263^259,260^ falls within this group. While displaying potential in inhibiting resistance in DTP cells, this anti-DTP model is hindered by a critical limitation: the lack of clinical application experience, leading to uncertainties regarding its efficacy, potential side effects, and harm to humans, as illustrated by ABT-263. Despite the initiation of clinical trials across various programs and the endorsement of numerous phase 2 and 3 trials,^261–265^ the presence of side effects or toxicity remains indeterminate, necessitating prolonged drug usage and further pertinent data. Furthermore, the adherence of cancer patients opting for clinical trials is often disparate, with a significant number unable to complete the trial, due to the lack of patience and courage. Nevertheless, this segment of preclinical drugs holds the highest potential for advancing treatment research and developing more precise anticancer resistance therapy.

### Anti-DTP drugs in traditional medicine mode

In today’s anticancer therapy landscape, traditional anticancer approaches still hold significant importance. However, the issue of drug-resistant cancer has previously cast doubts on the effectiveness of conventional therapy. This skepticism stemmed from a historical oversight regarding the reversible nature and partial memory capacity of DTP cells. Researchers, in the past, lacked confidence in traditional treatments due to the prolonged dormancy of drug-stimulated cancer cells in DTP, neglecting the possibility of their reactivation and vulnerability to the original drugs during the recovery phase, potentially leading to the destruction of transitional DTP cells. Therefore, it is not surprising that novel use for old drugs is being proposed. This new approach aims to redirect attention to drugs, whether with known anticancer properties or not, to investigate their relevance in combating DTP cells and to provide guidance on their usage in this context. Past extensive exploration into the properties, contraindications, and side effects of these drugs has been conducted, leading to their widespread adoption in treatments. For instance, disulfiram has exhibited efficacy in aiding cancer-resistant therapy by preserving redox homeostasis.^266^ Nonetheless, a notable challenge lies in the lack of clear dosages for many drugs selected for anti-DTP therapy, as effective doses often approaching toxic levels. Addressing these dosing uncertainties would require further extensive research—a daunting task that lies ahead. However, it is a piece of great news that expanding the application of this concept beyond oncology-focused drugs and conducting comprehensive screenings for potential anti-DTP therapy could pave the way for the future development of innovative treatment strategies.

### Anti-DTP drugs in Traditional Chinese Medicine assisted mode

The empirical cases of Traditional Chinese Medicine (TCM) contributing to the diagnosis and treatment of tumors indicate that traditional Chinese medicine plays a significant role in anticancer therapy,^267–269^ which cannot be overlooked.^270^ The emphasis on natural products in traditional Chinese medicine can aid in promoting the concept of anticancer treatment, appealing to cancer patients who opt for traditional Chinese medicine due to their preference for natural remedies over synthetic drugs. Natural products, primarily derived from natural bacteria and Chinese herbs, consist of antifungal and related components that offer diverse and intricate effects. While these natural products exhibit multifaceted drug efficacy, their precise influence on cancer cells and quantifiable impacts remain challenging to analyze. Nevertheless, notable examples, such as emodin’s ability to modulate reactive oxygen levels and disrupt the redox system equilibrium in cancer cells, highlight promising avenues for research.^271^ Despite the potential benefits, the inherent trace toxicity of many natural products complicates their application, necessitating strategies for mitigating adverse effects. Although the understanding of natural products remains limited, the vast array of components provided by nature offers abundant opportunities for exploration and discovery. Therefore, a comprehensive investigation of natural products represents a parallel approach to advancing anticancer treatment within the realm of traditional Chinese medicine.

Overall, it is hoped that such a categorization will provide methods and inspiration for the clinical management of DTP cells.

## Anticipated DTP: Challenges and prospects of anti-DTP therapy

The previous section provided a brief overview of the components involved in anti-DTP therapy. In the following section, we will delve deeper into the fundamental aspects of anti-DTP therapy and analyze its future directions and perspectives. The primary aim of anti-DTP therapy is to administer the treatment as necessary and feasible, while carefully weighing its benefits and risks, and being well-prepared to manage drug interactions, dosing regimens, and side effects.

### The necessary for anti-DTP therapy

When considering the necessity of treatments, it is essential to account for the ethical implications. In scientific research, emphasis is placed on cells, animals, and organ-like models, while clinical medicine prioritizes the treatment of patients. Thus, the significance of anti-DTP treatment in cell and animal experiments might differ from its application in cancer patients. In basic research, researchers can assert the necessity and meaning of anti-DTP treatment. However, in medical treatment, the acceptance of a medical intervention should not hinge on the question of necessity.

This is particularly pertinent in oncology where repetitive questioning of the necessity of treatments is trivial. Historically, surgical methods for tumors did not entail postoperative adjuvant chemotherapy or neoadjuvant therapy. Yet, presently, nearly all surgical interventions for solid tumors, particularly intermediate- and advanced-stage malignant tumors, stress the essentiality of postoperative adjuvant chemotherapy. This is because clinical evidence has demonstrated the efficacy of postoperative adjuvant chemotherapy in enhancing the outcomes of such tumors. It has been shown to improve the 5-year survival rate,^272,273^ as well as reduce the likelihood of cancer recurrence and metastasis^274^ following surgical treatment for these tumors.

Despite the lack of extensive clinical data on anti-DTP therapy, insights from in vitro experiments and organoid models suggest its potential to inhibit the development of drug resistance in cancer cells. Although one study by Yi Pu et al.^4^ mathematically supports the ability of anti-DTP therapy to lessen drug resistance, it is imperative to acknowledge that it is purely theoretical and requires validation through further clinical studies. Drawing from the mathematical analysis, it can be inferred that administering anti-DTP therapy after initial anticancer treatment can effectively alleviate drug resistance and tumor burden in the long term, making it an indispensable aspect of comprehensive anticancer therapy. The encouraging news is that an increasing number of relevant therapeutic studies are being conducted. For instance, Navitoclax,^265^ a clinical drug developed based on ABT-263, has shown promising results in combating drug resistance in DTP cells associated with drug-resistant lymphoma,^275^ EGFR-resistant NSCLC,^276^, and recurrent epithelial ovarian cancer.^277^ This serves as sufficient evidence to demonstrate the effectiveness of anti-DTP therapy in treating drug-resistant tumors. However, due to the limited availability of existing clinical trial data, widespread acceptance of anti-DTP therapy remains challenging. Therefore, further comprehensive research on anti-DTP therapy will be the focal point of the next era in addressing cancer resistance.

Furthermore, in the context of targeted therapy for NSCLC, many patients have had to undergo multiple rounds of changing targeted drugs due to the inadequacy of existing FDA-approved drugs in addressing multi-drug resistance.^278^ In the face of this challenge, anti-DTP therapy may be recognized as crucial in reducing the emergence of such drug resistance, thereby becoming a necessary intervention. It is hoped that the future availability of more extensive clinical data will be instrumental in establishing this necessity as a standard guideline.

### The feasibility for anti-DTP therapy

The feasibility of anti-DTP therapy is a significant concern given the current limitations in drug development. Even though previous research has identified several potential anti-DTP drug components, many of them are still in the preclinical stage, raising uncertainty about their effectiveness in the complex human body environment.^279^ While these components have exhibited the ability to inhibit DTP formation in vitro and target DTP cells for destruction,^41^ their efficacy in vivo is still uncertain due to potential challenges related to metabolic distribution and kinetic action. It is essential to maintain a balanced and objective approach when discussing the potential side effects and toxicity of anti-DTP drugs.

Despite these limitations, current evidence suggests that anti-DTP therapy may be a feasible and necessary approach for anticancer treatment.^33^ Currently, utilizing low toxicity or harmless novel use for old drugs and natural products^280^ represents a promising direction, even though they may not directly target the DTP cells. Nonetheless, their multifaceted effects can still have a significant impact and may be preferable for reducing side effects when used in integrative treatment.^281^

**Fig. 8:**
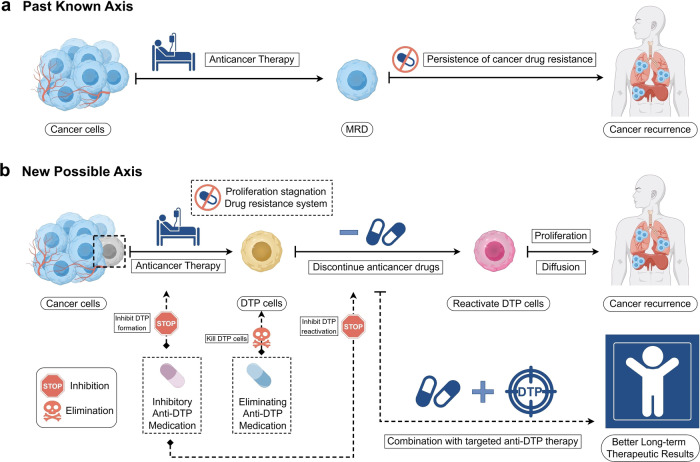
Anticancer Therapy Long-Term View. Past known axis (**a**) focuses on the drug resistance progress of minimal residual disease (MRD) after anticancer therapy and pursues to prevent the generation of MRD which leads to cancer recurrence. However, the emergence of a new possible axis (**b**) acknowledges the presence of DTP cells and reactivated one as the fundamental condition. Consequently, two new therapeutic schemes are proposed to simultaneously inhibit and eliminate DTP, thereby aiming for better long-term therapeutic results. (Drawn by figdraw)

In addition, the treatment of SCLC could be evidence, which involves targeted therapy with Niraparib^282^ after chemotherapy to reduce the tumor burden caused by the expansion of cancer cells that were not killed by chemotherapy.^283^ In the past, the understanding of MRD was more focused on targeting common cancer cells that were not completely removed. However, when combined with the existing analysis of DTP cells, it is possible that these surviving cancer cells have undergone non-genetic changes and now exist as DTP cells. It is important to consider this possibility when developing treatment plans. The long-term targeted therapy indirectly kills the repopulating ordinary cancer cells that have converted back from DTP cells. This indirect action has been proven to have therapeutic significance. Therefore, anti-DTP therapy that directly acts on DTP cells should be considered (Fig. 8). What’s more, the indirect long-term regimen of targeting ordinary cancer cells formed by DTP presents a hidden danger in terms of longer-term efficacy. This is due to the possibility of developing drug resistance when DTP is transformed into repopulating cells, which increases the risk of recurrence.^113^ Direct targeting of the DTP cells is necessary to ensure a lower risk of recurrence, emphasizing the need for anti-DTP therapy.

Furthermore, understanding and familiarity with the drug resistance mechanisms related to the DTP also provide meaningful support for the feasibility of anti-DTP therapy. These mechanisms are interconnected and complementary, allowing one to compensate for each other and maintain DTP cells’ homeostasis. However, this interconnectedness also renders the system vulnerable to targeted attacks that can disrupt the balance and lead to system collapse. Researchers have identified various potentially targeted basic mechanisms of DTP, such as molecular modifications like KDM5A^284^ and GAS6,^285,286^ regulation of pathways including Hippo^61^ and Wnt/β-catenin,^177^ and maintenance of redox homeostasis by components like GPX4^33^ and ALDH.^287^ Several in vitro studies targeting these mechanisms have yielded promising results,^238,288^ such as anti-DTP therapy targeting GPX4 has shown reductions in drug-resistant cancer cells,^8^ and inhibition of the mTOR pathway reversed DTP cells formation.^80^ Understanding the molecular modifications, signaling pathway regulations, and redox homeostasis underlying DTP cells suggests the feasibility of advancing from in vitro trials to clinical trials for validating anti-DTP therapy validation. Nonetheless, challenges remain, particularly in targeting resistance components in DTP cells while minimizing impacts on normal tissues. Despite these obstacles, the promise of anti-DTP therapy in cancer treatment underscores the importance of pursuing its development without delay.

### Utilization and prospects of anti-DTP therapy

As previously mentioned, anti-DTP therapy is significant. Therefore, its use is noteworthy. Similar to previous comprehensive oncology regimens, the potential benefits of anti-DTP therapy can be considered based on timing. The following outlooks can be initially considered: 1. Anticancer therapy combined with anti-DTP therapy: 1a) during the treatment period, or 1b) during the inter-therapeutic period; 2. Anticancer therapy followed by anti-DTP therapy: 2a) lethal or 2b) inhibitory.

The strategy of combining anti-DTP with anticancer therapy could be considered a promising approach. Direct combination of anti-DTP drugs during the therapeutic period offers the advantage of fusing anticancer and anti-DTP drugs. This approach can potentially enhance the efficacy of anti-DTP therapy without extending the patient’s hospitalization period, thereby ensuring improved quality of life and compliance. Compound Match Formulations,^289^ as a newer dosing regimen, are a good way forward for this treatment. Nonetheless, the potential drawbacks include unresolved concerns about drug interactions between existing antineoplastic drugs and anti-DTP, as well as the hepatic and renal loads resulting from the combined metabolism of the two drugs. Conversely, the inter-treatment period approach is viewed as more feasible for reducing the drug load and preventing delayed DTP formation. However, the potential impact of additional anti-DTP treatment on the patient’s body, such as the risk of infections, needs further investigation. Furthermore, the patient’s acceptance of the potentially more complex treatment regimen is another crucial aspect to be addressed. In summary, the role of an additional anti-DTP treatment with anticancer therapy provides a more considered approach to preventing DTP formation.

**Fig. 9:**
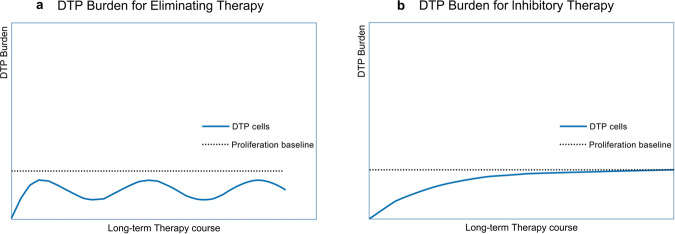
DTP Burden Forecast Trends. Periodic Eliminating therapy (**a**) involves administering drugs to DTP cells regularly to diminish the DTP burden before it surpasses the proliferation baseline, therefore ensuring it remains below this threshold. On the other hand, continuous inhibitory therapy (**b**) is focused on impeding the buildup of the DTP burden and ultimately steadying the quantity of DTP cells at a plateau that is beneath the proliferative baseline

Another perspective on anti-DTP therapy involves transforming long-term maintenance therapy following existing anticancer combination therapy. This transformation shifts the focus from targeting expanded MRDs to targeting their potential origin – DTP cells. Additionally, anti-DTP therapy offers various options due to the different mechanisms of action of the drugs. For instance, inhibitors targeting GPX4 can directly eliminate DTP cells, while drugs like Fluvastatin act in the tumor micro-environment to primarily inhibit DTP transformation.^8^ Consequently, the addition of anti-DTP therapy after anticancer therapy can involve regular killing of DTP, continuous inhibition of DTP, or a combination of both. In a regular killing regimen, the number of DTP cells undergoes waveform changes but remains below a baseline (Fig. 9), preventing extensive transformation to the proliferative state of ordinary cancer cells. On the other hand, continuous inhibition of DTP transformation may not reduce the basal number of DTP cells but aims to stabilize their presence at a plateau below the proliferative baseline (Fig. 9). Furthermore, a combination of both regimens may represent an optimal approach, provided that the side effects, toxicity, and economic implications of the drugs are considered.

In addition to the above considerations, researchers should further investigate the interactions between anti-DTP therapy and existing anticancer therapy. Anticancer therapy primarily target signaling pathways in cancer cells, while some anti-DTP therapy also affects signaling pathways. This raises questions about potential interactions between the two types of treatment. Moreover, traditional chemotherapy could theoretically be combined with anti-DTP therapy since chemotherapy targets active cancer cells while anti-DTP therapy targets dormant DTP cells. However, a key concern is whether the DNA damage caused by chemotherapy drugs may interfere with the molecular modifications targeted by anti-DTP therapy. Both anti-DTP therapy and anticancer therapy are complex treatments with multiple mechanisms. Identifying the effective mechanisms and determining whether they can synergistically enhance each other poses a challenging but crucial opportunity to gain deeper insights into DTP cellular mechanisms.

The dose planning of drugs is also a direction that needs to be explored. In the past, adequate doses, and regular and long-term anticancer treatment were considered the best course of action. It was believed that maximizing drug dosage could prevent the development of drug resistance, akin to treating conventional bacterial infections. However, the discovery of DTP cells has challenged this assumption by weakening the established connection between drug dosage and resistance in cancer. This, in turn, raises concerns about the increased risk of side effects without a corresponding decrease in drug resistance. Therefore, pursuing research into the optimal planning of drug dosages post-combined anti-DTP and anticancer therapy holds significant promise. Further investigations should focus on gradually reducing anticancer drug therapy while inhibiting DTP cell production to prevent drug resistance from emerging.

The management of drug side effects is a crucial yet often neglected aspect of treatment. Patients commonly find the side effects of traditional chemotherapy, including hair loss, nausea, vomiting, and marrow hemopoietic suppression, to be intolerable. Moreover, the liver toxicity, nausea, diarrhea, and other adverse effects associated with anti-DTP therapy can significantly compound the discomfort experienced by patients undergoing treatment. Therefore, it is paramount to emphasize the monitoring and mitigation of side effects in the combination of anti-DTP therapy and cancer treatment regimens. Effective management of drug side effects is essential to prevent adverse effects from compounding or interacting negatively in combination therapy. Strategies should focus on minimizing side effects, such as reducing liver damage through the use of medications with different metabolic pathways.

Certainly, the essential guideline for planning anti-DTP therapy involves individualization and specific analysis of the particular problem. Anyway, a promising area for future research lies in the integration of anticancer therapy and anti-DTP therapy to develop a new comprehensive treatment for cancer. Therefore, there is a hopeful anticipation for more DTP clinical research and the development of targeted drugs for anti-DTP therapy.

## Conclusion and perspective

The development of new forms of DTP cells derived from ordinary cancer cells has provided researchers with a deeper understanding of the comprehensive drug resistance mechanisms in cancer cells. This advancement goes beyond simply examining single gene mutations or random changes, allowing for a more thorough investigation into the array of drivers behind non-genetic mechanisms. This new perspective on cancer resistance challenges conventional notions and offers an opportunity to reshape the prevailing pessimism associated with relapsed and metastatic cancer. By shedding light on the comprehensive drivers of drug resistance, this innovative approach can drive progress in combating resistant cancer. However, the multi-faceted interactions of DTP cells, being non-genetic in nature, present a complex web of mechanisms contributing to drug resistance. It is evident that the diversity and interaction complexity among these mechanisms require further exploration to steadfastly advance our understanding in this area.

This review encompasses a brand range of the concept of DTP, the potential transformation process of DTP, and the comprehensive mechanism of drug resistance, and emphasizes the essential difference between the old concept of various related cancer-resistant cells and DTP cells, which exhibits a reversible inert specialization in response to external stimuli. By analyzing the common characteristic changes during DTP transformation, such as cell-cycle restriction, embryonic-like diapause, stem-like phenotype, and reversibility of transformation, it is clearly recognized that cancer cell transformation into DTP cells poses a significant challenge in anticancer therapy. Understanding the formation process of DTP cells and the development of related drug resistance mechanisms has led researchers to realize that DTP cells, although a stubborn component of cancer drug resistance, are not insurmountable obstacles. The interaction of molecular modification regulation, signaling pathways and transcription regulation, tumor micro-environment regulation, metabolic reprogramming regulation, and redox systems regulation behind the drug resistance mechanism of DTP cells has been elementary investigated. Continuous studies are identifying various targeted strategies for destroying corresponding drug resistance mechanisms. The exploration of numerous targets, in conjunction with the clinical experiment-oriented medicine model, traditional evidence-based medicine model, and traditional Chinese medicine-assisted medicine model, has led to the investigation of preclinical drugs, novel use for old drugs, and natural products within these medical paradigms to discover potential anti-DTP therapy. This exploration will serve as a key component of long-term comprehensive anticancer treatment in the future, offering new therapeutic benefits for patients with relapsed or metastatic middle and advanced cancer.

For future research, we anticipate exploring the following aspects further:

1. Existing DTP studies primarily rely on in vitro cell tests, with some probes like Mex3a identified as potential markers^15^ for DTP detection in basic experiments. However, current research lacks specific indicators that could offer insights into organoids or cancer patients. To address this gap, conducting in-depth investigations into the expression patterns of molecules associated with DTP in conjunction with analyzing pathological tissues from recurrent cancer patients holds promise in providing crucial evidence for early clinical identification of DTP transformation during anticancer therapy.
2. Analyzing the essential differences underlying the characterization of ordinary cancer cells before transformation, transformed DTP cells, and cancer stem cells of corresponding cancer species through single cell-seq offers a more fundamental perspective. These precise analyses will directly and indirectly guide subsequent DTP studies by thoroughly separating DTP from special cells such as cancer stem cells with some plausible correlation. Additionally, the analyses will reveal how these cells emerge and exist at the micro level, shedding light on their impact on the persistence of macroscopic DTP cells.
3. Data collection and peak recording of DTP cells transformation curves through different drug inductions, stepped killing of drug concentrations, and corresponding DTP cells transformation curves is a crucial undertaking that demands significant effort. The induced generation of DTP cells, which is typically a minuscule fraction of the residual population following drug resistance therapy, exhibits extremely low proliferation activity and diminished responsiveness to external stimulation. The resulting scarcity at the cellular level poses challenges for cytological experiments aimed at passage, proliferation, and culture. While existing DTP research primarily focuses on reactivating DTP cells activity through drug holidays, there is a pressing need to advance the field by achieving stable induction of DTP cells production and expanding the cell population to a level conducive for in-depth studies. Enriching DTP cells sufficiently to support investigations into reactivation and proliferation dynamics under sustained external drug stimulation is key for furthering understanding in this area. Current studies have largely overlooked the importance of DTP cells enrichment and external competition due to intrinsic limitations in DTP cells quantity and challenging conditions for amplification in vitro tests. In clinical contexts, there exists a potential scenario where the quantity of DTP cells surpasses a critical threshold due to improved nutritional status and reduced immune factors, potentially lead to non-drug induced DTP reactivation amidst continued drug exposure. This shift in focus towards enriching and understanding DTP cells dynamics under varying conditions represents a critical advancement that will significantly contribute to the field of DTP research.
4. At present, the study of more specific and precise drug resistance mechanism targets represents a key advantage in DTP research. However, the challenges presented by the stable structure created through the interconnection and synergy among various drug resistance mechanisms should not be overlooked. By conducting a comprehensive circular analysis starting from a molecular modification regulation standpoint, researchers can gain insights into breaking through the existing resistance barriers in DTP. This approach involves examining the relationship between the influence of molecular modification regulation on upstream and downstream pathways, the proportion of components in the tumor micro-environment following overexpression or knockdown, variations in nutrient consumption and energy metabolism, and alterations in redox stability tolerance. Through this process, a better understanding of anticancer drug resistance can be established. The ultimate goal is to selectively eliminate essential components of the DTP resistant system without disrupting the body’s homeostasis, thereby significantly advancing the treatment of DTP-related cancer resistance.

In recent years, there has been a growing emphasis on research and exploration into cancer drug resistance, with DTP-related studies playing a significant role in this endeavor. DTP cells, which emerge as a result of cancer cells undergoing specialized adaptations in response to drug exposure, represent a distinct subgroup of cancer cells that exhibit pivotal non-genetic characteristics in comparison with those seen in various cancer cell lineages. Despite their diverse origins, DTP cells undergo a series of dynamic transformations that can be intricately studied. This detailed examination of DTP cells not only offers valuable insights into cancer drug resistance at a fundamental level but also provides a method for addressing drug resistance in practical applications. By bridging the gap between basic and applied research, the comprehensive investigation of DTP cells holds promise for advancing our understanding of cancer drug resistance and, ultimately, enhancing patient outcomes in oncology.
